# An explorative study of factors associated with treatment in specialized mental health care centers among GP patients in Norway

**DOI:** 10.1186/s12913-021-06982-4

**Published:** 2021-09-13

**Authors:** Ole Gunnar Tveit, Torleif Ruud, Ketil Hanssen-Bauer, Ole Rikard Haavet, Ajmal Hussain

**Affiliations:** 1 Division of Mental Health Services, Akershus University Hospital, Lørenskog, Norway; 2 Institute of Clinical Medicine, Faculty of Medicine, University of Oslo, Oslo, Norway; 3 Department of General Practice, Institute of Health and Society, Faculty of Medicine, University of Oslo, Oslo, Norway

**Keywords:** Psychiatric specialist services, Mental health care, Health care utilization, Norway

## Abstract

**Background:**

Effectiveness and efficiency are part of the quality of care for mental health problems, and treatment should thus be performed at the right level of care. Norwegian guidelines specify which patients should be given priority for treatment in specialized mental health care (SMHC) centers, but there is a lack of agreement on which patients should actually receive SMHC. In this study we wanted to examine what factors (patient and GP characteristics) were related to GP patients who received treatment in SMHC centers.

**Methods:**

In this retrospective cohort study, we looked at 12 months of data from electronic health records from six GP and SMHC centers of hospitals in the catchment area. We included all patients who had been treated at any of the GP centers during the 12-month period (N=18032). We fit a generalized linear mixed model to explore which factors were related to patients receiving treatment in SMHC centers. Further exploration was performed to study the effects of gender and contact frequency.

**Results:**

We found that 4.6% of all GP patients and 18.4% of the GP patients with a mental health problems were treated in SMHC centers. There were more women than men among the GP-patients (56% vs 44%) and in SMHC centers (55% vs 45%), women with mental health problems were more severely ill than men. However, after adjusting for other factors men were more likely to be treated in SMHC centers (OR: 1.44). Patients with frequent GP contact were more likely to be treated in SMHC centers. The GP characteristics age, gender and specialization did not relate to patients receiving treatment in SMHC centers.

**Conclusions:**

Men were more likely to be treated in SMHC centers than women, which may imply that they have different thresholds for entering SMHC centers. GP characteristics were not related to receiving treatment in SMHC centers. More specific knowledge is needed to determine whether men and women currently receive treatment at the lowest possible level of care.

## Background

Treating patients at the optimal level of care is not straightforward. Several definitions of quality in health care include the dimensions effective and efficient [1, 2]. Based on these dimensions, patients with less severe or easier to treat mental health problems (MHPs) should receive their mental health care at the primary level, e.g. from a general practitioner (GP). Care at the specialized level should be reserved for patients with more severe MHPs. An important mechanism to ensure that patients are treated at the optimal level of care is that the patient has to be referred by a GP to have access to specialized mental health care (SMHC).

The Norwegian guidelines for referrals to SMHC state that moderate or severe mental disorder should be given priority, while milder problems should not [3, 4]. This does not mean that all patients with moderate and severe disorders enter or are referred to SMHC centers. Some may respond well to treatment in primary care so that neither the patient nor their GP sees any need for referral. A study found low agreement when comparing the judgments of 42 SMHC intake teams assessing the same referrals [5]. A large variation is reported in acceptance rates of referrals from GPs among Norwegian community mental health centers (part of SMHC) [6]. These findings indicate a low agreement between professionals at the two levels of care in regard to the criteria for admittance to SMHC centers and a difference in the competencies of different GPs.

Every person in Norway is provided a regular GP, equivalent to a family physician. Only 0.2% of the population has chosen to remain unassigned to any GP [7]. GP and SMHC centers are financed by different sources. Most GP centers are financed jointly by boroughs or municipalities (per capita) and states (tariffs), while SMHC centers are run by governmental health trusts. In addition, the patients copay small amounts for the consultations both at the GP centers and at the SMHC centers. Sixty-eight percent of the patients who are treated in SMHC outpatient clinics in Norway are referred by their GP [8], while 24% are referred or transferred from another SMHC unit. Other than the severity of the mental health problem, it is not well studied what contributes to whether patients are treated in SMHC centers.

There has been some interest in whether the characteristics of the patient could be related to receiving treatment. Studies have found lower help-seeking behavior among men than among women in general [9], reduced chance of GPs registering an MHP after seeing male patients compared to after seeing female patients [10], and reduced probability that men would be treated in psychiatric outpatient clinics compared to the probability among women [11]. Seeking help for MHPs also seems to increase with age (especially for men) [9]. Possible gender interactions (between GP and patient) have not been studied in regard to referrals to SMHC centers. However, a study found that male patients had more use of short-term sick leave for any reason than female patients but only among patients of male GPs [12]. Studies have consistently found that married patients are more seldom referred to SMHC centers than unmarried patients [13]. Additionally, patients with frequent contact with outpatient specialized somatic clinics had an increased use of SMHC [14]. However, the relationship between the utilization of primary health care and of SMHC has not been studied extensively among GP patients.

Another vein of interest is whether characteristics of the GPs are related to whether patients are treated in SMHC centers. One study using patient actors found that male GPs referred their patients to SMHC centers more often than female GPs, whereas neither the GPs’ specialization nor age were associated with whether the patients were referred or not [15]. However, a study from Norway found that female GPs were more likely to refer patients to specialized care, including somatic and mental health care, than male GPs, while specialized GPs (family medicine) were less likely to refer patients than nonspecialized GPs [16]. These conflicting results warrant further investigation.

### Aims

In this study we wanted to examine what factors were related to GP patients who received treatment in SMHC centers. More specifically we wanted to explore the following questions: 1) Is the patient’s gender related to whether they are treated in an SMHC center? 2) Are the patients’ contact frequencies with the GPs related to being treated in an SMHC center? 3) Are the GP characteristics age, gender, and specialization associated with patients getting treated in SMHC centers?

## Methods

### Design and setting

In a retrospective cohort design, we included a sample of patients who had undergone one or more consultations between the 5th of May 2014 and the 4th of May 2015 with GPs at six GP centers in the Groruddalen area in Oslo, Norway. We collected data about these patients from the electronic patient records at the GP centers, the SMHC center at Akershus University Hospital (Ahus) and the welfare services. This Groruddalen area in Oslo is characterized by a low socioeconomic level, with many inhabitants having an immigrant background (first to third generation).

### Participants

We included all patients older than 16 years and younger than 66 years on the 5th of May 2015 who had undergone a consultation at one of the six included GP centers during the 12-month period before the 5th of May 2015.

We excluded patients who lacked a personal identification number in their electronic records and patients who did not have an assigned GP at the center.

### Variables

Data for all variables were collected by electronic transmission from the electronic patient records of the six GP centers and Ahus, and from the client database at the Norwegian Labor and Welfare Administration (NAV).

From the electronic patient records at the GP centers we collected data on gender, year of birth, name of the consulting GP, number of consultations during the 12-month period, and the tariffs used. Consultations were specified as direct or indirect consultations. A consultation was direct if the patient met with any GP at the GP center during the consultation; otherwise, the consultation was indirect. Examples of indirect contact are contacts via telephone or letters contact with patients or other services, GPs filling electronic prescriptions or GPs completing required forms. We collected information on GP age, gender and specialization from the Norwegian Health Personnel Registry [17].

From all the clinical departments within the SMHC center at Ahus we collected data on any consultations and admissions during the 12-month period.

In addition, we collected data about marital status and immigration status from the NAV registers for all the patients.

To adjust for patients’ mental health problem severity, we used the GPs’ diagnostic coding. We collected data on the reason for the consultation registered by the GP using the International Classification of Primary Care-Version 2 (ICPC-2) [18]. This classification categorizes the reasons using either problem codes or diagnostic codes, with diagnostic codes being more specific and implying more severe illness. We grouped patients according to whether they received: 1) neither mental health problem codes nor mental health diagnostic codes, 2) at least one mental health problem code and no mental health diagnostic codes or 3) at least one mental health diagnostic code during the 12-month period.

#### Validating the severity score

This three-level variable was validated against the Clinical Outcomes in Routine Evaluation (CORE-10) measure of psychological distress, a measure developed for assessing common mental health problems in a primary care setting [19]. The CORE-10 measure was completed by a subset (n = 850) of the included patients. Patients were recruited during the final two weeks of the 12-month period. All patients present at daytime in the GP centers waiting rooms were asked if they could fill out a form containing CORE-10. Forms and return mailboxes were also set up so patients could fill out forms and return them in the evening. Response rates were not recorded. The subset was representative of the larger group, except that the subset had more contacts and a lower proportion of immigrants.

The two measures (MHPs as coded by the GP and the CORE-10 score) have a moderate Spearman correlation (*r*_s_=.39,*S*=26,079,954.92,*p*<.001). The mean CORE-10 score among patients with no MHP-related codes was 9.52 (low-level problems), patients with at least one code for mental health problems/symptoms (but not diagnosis) had an average CORE-10 score of 13.22 (mild psychological distress) and patients with at least one code for mental health diagnosis had an average CORE-10 score of 17.57 (moderate distress).

### Statistical analyses

We used a mixed model (multilevel) logistic regression to explore our research questions. GP centers and GPs were included with random intercepts in the model. Contact with SMHC centers during the 12-month period (yes/no) was used as the dependent variable. The model was estimated using an unstructured covariance matrix. We calculated odds ratios (ORs) with confidence intervals for all explanatory variables. For all analyses, a significance criterion of *α*=.05 was used. Factors in the main model were tested for multicollinearity by the variance inflation factor [20]. Data were prepared and analyzed using R [Version 3.6.2; [21]] and SPSS [Version 25.0; [22]]. We used the following explanatory variables for the mixed model logistic regression: Gender, Age, Married (yes/no), Immigrant (yes/no), Direct Contacts (number of), Indirect Contacts (number of), Mental Health Symptom (but no diagnosis; yes/no), Mental Health Diagnosis (yes/no), GP age, GP gender and the interaction between patients gender and GPs gender.

In the follow-up exploratory analyses, we used linear regression to study differences in CORE-10 scores according to gender and differences in contact frequency according to gender. To further explore whether immigration related to the gender effect, we split our study sample into immigrants and nonimmigrants, and analyzed these two samples with the main regression model. We also estimated two interaction models for follow-up analyses. We started with the main model and then added interaction with all factors for gender and for direct contacts (a total of 21 factors in both models). Welch two-sample t-tests were used to study differences in contact frequency for patients within/outside of SMHC centers.

## Results

During the 12-month period, a total of 18410 patients were included from the six GP centers. We excluded 10 of the patients because of missing personal identification numbers, and we excluded 368 of them because of missing GP identification. Of the included patients (*N*=18032), we found that 835 patients (4.60%) had received SMHC services at Ahus.

We identified 47 different GPs for the included patients. These GPs were on average 45.4 years old (range: 27 - 70) and 62% female. Seventy-two percent were specialists in family medicine. The GPs treated an average of 384 (range: 2 - 927) patients and performed an average of 2377 (range: 2 - 6233) consultations.

Among all included patients, 4.60% received treatment at SMHC centers. Among the patients who the GPs had coded as having a mental health symptom or diagnosis, 18.6% were receiving treatment at an SMHC center. Furthermore, 21.4% of the patients in contact with SMHC centers were not coded by a GP as having an MHP. Among all patients, 19.7% were coded by GPs as having an MHP. Characteristics of the sample are shown in Table 1.

**Table 1:** Characteristics of the sample (N = 18032) according to contact with the specialized mental health care (SMHC) center

	Level	Not treated in SMHC	Treated in SMHC
n (%)		17197 (95.4)	835 (4.6)
Gender [n (%)]	Female	9664 (56.2)	461 (55.2)
	Male	7533 (43.8)	374 (44.8)
Age [Mean (SD)]		40.3 (13.7)	37.9 (13.3)
Married [n (%)]	No	9163 (53.3)	648 (77.6)
	Yes	8034 (46.7)	187 (22.4)
Immigrant [n (%)]	No	7840 (45.6)	321 (38.4)
	Yes	9357 (54.4)	514 (61.6)
Direct Contacts [Median [min, max]]		3 [0, 98]	5 [0, 80]
Indirect Contacts [Median [min, max]]		2 [0, 107]	5 [0, 234]
Total Contacts [Median [min, max]]		5 [1, 124]	12 [1, 246]
Mental health problem [n (%)]	No	14299 (83.1)	179 (21.4)
	Symptom	1571 (9.1)	132 (15.8)
	Diagnosis	1327 (7.7)	524 (62.8)

*Note.* Direct contact: consultation with a GP at a GP center. Indirect contact: any other contact with the GP center, GP filling out forms, letter of referral, etc. Total contacts: the sum of direct and indirect contacts. Mental health problems were symptoms or diagnoses if a GP coded that the patient had any mental health symptoms or diagnoses during the 12-month period.

### Main regression model

We found 44% higher odds of treatment in SMHC centers among male patients (*OR*=1.44 (95% CI: 1.14 – 1.83), *p*=.003). We found a negative association between the age of the patient and treatment in SMHC centers (*OR*=0.98 (95% CI: 0.97 – 0.98), *p*<.001). The odds for treatment in SMHC centers decreased 2% per year as the patients’ age increased. Married patients had lower odds of treatment in SMHC centers (*OR*=0.48 (95% CI: 0.40 – 0.59), *p*<.001) than unmarried patients. Being an immigrant in general had no significant association with treatment in SMHC centers (*OR*=1.02 (95% CI: 0.86 – 1.22), *p*=.791), although the unadjusted effect was significant (*OR*=0.72 (95% CI: 0.62 – 0.84), *p*<.001).

We found a positive relationship between GP contact frequency and treatment in SMHC centers (*OR*=1.03 (95% CI: 1.01 – 1.04), *p*<.001). The odds of receiving treatment in SMHC centers increased by 3% for each additional direct consultation with a GP. We also found increased odds of treatment in SMHC centers associated with higher indirect GP center contact frequency (*OR*=1.04 (95% CI: 1.03 – 1.05), *p*<.001). Here, odds of treatment in SMHC centers increase by 4% for each additional indirect consultation with the GP center.

We found significant associations between patients’ treatment in SMHC centers and GPs marking the patients’ reasons for visiting as being related to mental health symptoms or diagnoses. There was a positive association between mental health related ICPC symptoms and SMHC treatment *OR*=5.95 (95% CI: 4.69 – 7.55), *p*<.001. When GPs marked the reason as a mental health diagnosis, the association was stronger (*OR*=25.72 (95% CI: 21.22 – 31.18), *p*<.001).

We found no significant effects of GP age, gender or specialization in either adjusted or unadjusted models. We found no evidence of an association of patient/GP gender interaction with treatment in SMHC centers.

All regression coefficients for the main model are shown in Table 2. The clustering structure of the model (patients nested in GPs, nested in GP centers) explained 3% of the variation in who received treatment in SMHC centers (intraclass correlation coefficient; ICC = 0.03). The model had a marginal *r*^2^ of 0.34 (variance explained by the fixed part of the model) and a conditional *r*^2^ = 0.35 (variance explained by the fixed + the random parts of the model).

**Table 2:** Regression analysis (generalized linear mixed model) of factors associated with treatment in specialized mental health care (SMHC) centers

	Unadjusted			Adjusted		
	OR	CI (95%)	p	OR	CI (95%)	p
Male (ref: Female)	1.04	0.90 – 1.20	0.573	1.44	1.14 – 1.83	0.003
Age	0.99	0.98 – 0.99	<0.001	0.98	0.97 – 0.98	<0.001
Married (ref: Not married)	0.33	0.28 – 0.39	<0.001	0.48	0.40 – 0.59	<0.001
Immigrant (ref: Not immigrant)	0.72	0.62 – 0.84	<0.001	1.02	0.86 – 1.22	0.791
No. of Direct Contacts GP	1.10	1.09 – 1.12	<0.001	1.03	1.01 – 1.04	<0.001
No. of Indirect Contacts GP	1.07	1.07 – 1.08	<0.001	1.04	1.03 – 1.05	<0.001
Mental Health symptoms only	1.89	1.55 – 2.29	<0.001	5.95	4.69 – 7.55	<0.001
Mental Health diagnosis	20.40	17.49 – 23.80	<0.001	25.72	21.22 – 31.18	<0.001
GP age	1.00	0.99 – 1.02	0.675	1.00	0.99 – 1.01	0.854
GP is specialist (ref: GP not specialist)	1.08	0.76 – 1.52	0.682	1.09	0.76 – 1.56	0.642
GP is male (ref: GP is female)	1.05	0.78 – 1.41	0.770	1.09	0.80 – 1.48	0.589
Gender interaction (Male:GP male)	0.84	0.63 – 1.12	0.237	0.84	0.61 – 1.16	0.282

*Note.* Mental health symptoms only refers to whether a GP has coded a mental health problem but does not refer to a diagnosis of the patient. Mental health diagnosis refer to when a GP has coded a mental health diagnosis (with or without additional symptom code). Gender interaction is the additional effect when both the patient and GP are male.

### Exploring the possible gender effect

As seen in Table 3, men had slightly lower self-rated psychological distress scores on CORE-10 than women (filled out by a subset of N = 850 patients). Among all patients, we found that the proportion of patients who received a mental health-related diagnostic code from a GP was 21% among women and 18% among men.

**Table 3:** Regression analysis: Gender effect on self-rated psychological distress (CORE-10) controlled for treatment status (N = 850)

Predictor	*b*	95% CI	*t*(633)	*p*
Intercept	11.02	[10.38,11.65]	33.93	<.001
GenderM	-1.13	[−2.22,−0.04]	-2.03	.042
ContactSMHC	6.89	[4.87,8.90]	6.70	<.001

*Note.* Self-rated psychological distress measured by CORE-10. SMHC: Specialized mental health care.

We also noted less contact with GPs among men than among women. This applies to both direct contacts with GPs (*b*=−1.11, 95% CI [−1.24,−0.99],*t*(18398)=−17.76,*p*<.001) and indirect contacts with GPs (*b*=−0.95, 95% CI [−1.14,−0.77],*t*(18398)=−10.07,*p*<.001). Similarly, we found that men had a lower odds ratio of receiving a diagnostic code from a GP than women (*b*=−0.19, 95% CI [−0.29,−0.09],*z*=−3.75,*p*<.001). The gender differences regarding symptom codes, however, were minimal (*b*=−0.01, 95% CI [−0.02,0.00],*t*(18398)=−2.23,*p*=.026).

The effects of gender seem to interact with immigration. We split the patient population according to immigration status (yes/no) and estimated the main regression model in both groups. For nonimmigrants we found a similar effect as found in the main model (*OR*=1.49 (95% CI: 1.11 – 2.01), *p*=.009). When estimating the model with immigrants only, however, the gender effect was not significant (*OR*=1.35 (95% CI: 0.92 – 2.00), *p*=.129).

Further, we estimated a model similar to the main model, but with added interactions effects between gender and each of the other factors (21 factors in total). The only significant interaction effect in this model was between gender and age (OR = 1.02 (95% CI: 1.01 - 1.03), p = 0.004). With this model, we thus find that womens’ odds for treatment in SMHC centers decreased 3% per year, while men’s odds for treatment in SMHC centers decreased 1% per year.

### Exploring the association between contact frequency and SMHC treatment

Among all the GP patients, patients with an MHP had significantly more direct contacts with a GP (*ΔM*=2.96, 95% CI [2.77,3.15],*t*(4,349.78)=30.25,*p*<.001), and more indirect contacts with a GP (*ΔM*=3.53, 95% CI [3.21,3.85],*t*(4,124.06)=21.68,*p*<.001). We found a small correlation between contact frequency and psychological distress using Spearman correlation (*r*_s_=.26,*S*=31,582,975.29,*p*<.001 for direct contact and *r*_s_=.22,*S*=33,465,282.82,*p*<.001 for indirect contact). From our interaction model with direct contacts, we see as expected that the interaction between direct contacts and getting a mental health diagnosis is significant (OR = 1.15 (95% CI: 1.08 - 1.21), p <0.001). For the interaction between GPs’ gender and direct contacts (OR = 0.97 (95% CI: 0.94- 1.00), p = 0.0.23), there is a weaker association between direct contacts and being in SMHC when the GP is male than when the GP is female. For the interaction between patient gender and direct contacts (OR = 1.04 (95% CI: 1.01 - 1.07), p = 0.009), male patients had a stronger association between direct contacts and being in SMHC than women.

## Discussion

We found that there were relatively few patients receiving treatment in SMHC centers (4.60%) among all the GP patients. When looking at those with an identified MHP (coded by a GP), only 18.4% received treatment in SMHC centers. However, patients with an identified MHP (symptom or diagnosis) had significantly increased likelihood of being treated in SMHC centers. Patients who had frequent contact with their GP (both direct and indirect contact) also had an increased likelihood of being treated in SMHC centers. There were more women than men among the GP patients; however, when adjusting for other factors, men were more likely to be treated in SMHC centers. The likelihood of being treated in SMHC centers reduced with age, and for patients who were married. The GP characteristics age, gender and specialization did not relate to receiving treatment in SMHC centers.

To our knowledge, this is the first study to address factors that may be of importance in the referral and decision-making process regarding receiving treatment in SMHC centers; this study used several data sources and followed the patient’s path from the GP office to the SMHC center. Among GP patients, up to 20% had visited the doctor’s office within 12 months due to mental health problems. These findings reinforce the impression from general practice that a minority of patients who have psychological problems are receiving treatment in SMHC centers. Slightly more than half of the participants in this study sample were immigrants. The high number of immigrants participating was expected as the region where the GP centers were located has a high proportion of immigrants [23]. The main strengths of this study are nevertheless the large population included (N = 18400), that the population consisted of all patients (16 to 66 years) of the GP centers, and that we have linked data on individuals from different sources.

Men were more likely to be treated in SMHC centers than women after adjusting for contact frequency and symptom severity (according to GP diagnostic coding). Unadjusted scores, however, showed no gender difference in the likelihood of being treated in SMHC centers. One possible explanation is that women had more severe mental problems than men. This is supported by our follow-up analysis, where we found that men, compared to women, had slightly lower scores of self-reported psychological distress, fewer contacts with GPs, and lower odds of receiving a diagnostic code from a GP. This is consistent with the reduced help-seeking behavior seen among men [9, 10]. If women’s likelihood of receiving treatment in SMHC centers corresponded to their somewhat higher illness severity, we would see a gender difference (women more likely than men to be treated in SMHC centers) in the unadjusted analyses. Our finding, conversely, seems to imply that men receive treatment in SMHC centers while being less severely ill than women (on average). This could indicate that there is a lower threshold of illness severity for entering SMHC centers for men than for women. It does not necessarily imply unfair provision of health services, however. Even if women in treatment at SMHC centers have more severe illness than men in SMHC on average, the men could still be within the criteria described in the guidelines for who should be prioritized for entry into SMHC centers [3].

Another possible reason for the gender effect is that men could have been in greater need of specialist services because the situation, such as work and family, required it and thus were referred even if less severely ill. We did not, however, find a significant interaction between gender and marital status in our follow-up analyses. Treatment in specialized care may also reflect men’s desperation and desire for rapid recovery [24], or there could be a gender difference in how severe patients rate their problems. Another possibility is that GPs to a greater extent were able to address the needs of female patients than male patients, which thus leading to a gender difference in who was referred to SMHC. In addition, there was a high proportion of immigrants in our study, and immigrant women are usually found to have a higher risk of MHPs than immigrant men [25, 26]. In our follow-up analyses, however, the gender effect was not significant when looking at immigrants only, indicating that the gender effect is not driven by increased risk among female immigrants. In our follow-up analyses, we found that although both men and women have a lower probability of being treated in SMHC centers as they get older, men do so to a lesser extent. This could mean that the mechanisms that contribute to the observed gender effect, those discussed above or others, do so in a more prominent way for older patients. Nonetheless, more research should be performed to better illuminate why men seem to have a higher likelihood of being treated in SMHC centers.

As expected, there was a strong association between GPs’ coding for the reason for patients’ consultations as an MHP and the patient being treated in an SMHC center. The GP coding was also consistent with patients’ self-reported psychological stress. Furthermore, a significant proportion of patients (21%) treated in SMHC centers did not receive an MHP code at any time during the 12-month period. One reason may be that the SMHC center, and not the GP, treated the mental health problems. Some patients may have stopped seeing their GP after receiving treatment at the SMHC center or only saw their GP for other issues. Some patients who were referred by the GP before the 12-month of data collection period were treated an SMHC center, not by the GP, for an MHP during the 12-month period. Patients who have had longer inpatient stays usually do not see their GP for any MHP during the stay. People who were in more frequent contact with their GP had an increased likelihood of being treated in an SMHC center. This is consistent with our finding that GP patients with an MHP had more direct and indirect contact than patients without MHPs. Some nuances appear in our follow-up interaction models. Male patients have a stronger association between direct contacts and being in SMHC than female patients, while the association was weaker if the GP were male than if the GP were woman. These two effects may have canceled each other out in our study, and may explain why we did not observe the patient/GP gender interaction seen by others [12]. These differences are small, but may contribute to the observed gender effect.

Ledoux, et al. [14] suggested possible mechanisms that may underlie the association between health care utilization and treatment in SMHC centers: 1) patients with more severe somatic illness could be more distressed and in need of mental health care, 2) increased contact could inform the health provider with a deeper understanding of the patients’ problems, which could increase the detection of mental health problems, 3) health providers may be frustrated by frequently appearing patients and may push harder for referral and 4) more dependent patients may be more open to getting support from additional health providers. In addition, we suggest 5) that patients who engage in more help-seeking behavior with their GP may have a higher likelihood of contact with any health care than those with less help-seeking behavior. The mechanisms described above may contribute to the observed association between direct and indirect contacts and patients’ treatment in SMHC centers.

The characteristics of GPs were not associated with patients receiving treatment in SMHC centers, in contrast to the findings of Ringberg, et al. [16] and Kravitz, et al. [15]. We had a larger sample than the other studies, but also measured a different outcome. Both Ringberg et al. [16] and Kravitz et al. [15] examined referrals sent from GPs, while we studied which patients had received treatment. Our design may introduce two additional mechanisms: 1) the intake teams in SMHC units filter referrals, where some of the referrals sent by GPs are rejected, and 2) some patients in our study may have entered SMHC centers by other means than referrals sent by GPs. These two mechanisms may counteract a possible effect of GP characteristics seen in other studies. Patients who are referred but not in need could be rejected, some in need can still be rejected and patients in need who are not referred may enter SMHC centers by other means. Another possible explanation for the differences may be the context. The Kravitz, et al. [15] study was performed in the US, while the present study was performed in Norway. The observed differences could be a result of differences in the GP’s role or education, health service structures, health service policy or cultural differences. As the Ringberg et al. [16] study was performed in Norway contextual differences from the present study should be smaller. Their study included referrals for both somatic and psychiatric services, with psychiatric referrals constituting only 6% of all the referrals. It is possible that their observed differences are not valid for psychiatric referrals.

### Limitations

We adjusted for patients’ mental health problem severity according to the GPs’ diagnostic coding, but these codes do not fully cover the actual severity of the condition of the patient. As discussed above, approximately one-fifth of patients who are treated in SMHC centers do not receive a diagnostic code related to mental health when visiting their GP; this group will be composed to false negatives when using the GPs’ coding of MHP as an indicator of treatment in SMHC centers. The indicator will also be inaccurate in cases where a patient has an MHP but it is not known to the GP. There could also be cases of false positives: if patients have been treated for an MHP and fully recovered, coding of consultations could still include the diagnosis. Nevertheless, these codes will be reflective of the GP evaluation of patients, and they provide an adequate indication of severity.

Our sample is biased in different ways, some of which are unknown to us. Most patients are assigned to a GP in the area where they live, and the GP centers in our study are all located in the Goruddalen area of Oslo. This area is characterized by a low socioeconomic level and by many inhabitants having an immigrant background (both refugees and labor immigrants) compared to other areas of Oslo. The six included centers are located in areas that may have local variations in these variables. From the model estimation we find that very little of the variation in the outcome can be attributed to which of these GP centers a patient belongs to. Nevertheless, by including all patients from the included centers we did not have a systematic selection bias based on biased response.

We know that the discovery of MHPs depends on several factors. The patients or the patients’ relatives have to detect a problem and decide that it is relevant to contact a GP for this problem. This decision is impacted by the patients’ needs, beliefs and views on mental health. On the GP side, there is also a judgment regarding the patients’ presentation of symptoms and cultural practices of how and what is coded. The GP will probably, among other actions, determine the relationship between the patient’s mental dysfunction and the patient’s coping before referring them to a SMHC. In the same way, receiving treatment in SMHC centers also depends on several factors. Overall, there are many possible mechanisms that make it difficult to predict whether a patient with any given characteristics will receive treatment in an SMHC center.

## Conclusion

Patients with MHPs had higher contact frequency with GPs than other patients, and patients who had higher contact frequency with their GP (both direct and indirect contact) had an increased likelihood of being treated in an SMHC center. Some of the increased contact may be attributed to collaboration between GPs and other service providers. In our sample, women had more severe MHPs than men, but there was no gender difference regarding who received treatment in SMHC centers. When adjusting for illness severity, men were more likely to be treated in SMHC centers. Overall, it would seem that men were less severely ill when being treated in SMHC center. This implies that men have a lower threshold for entering SMHC centers than women. If so, this could mean that health care is distributed unfairly. More specific knowledge is needed to determine whether men or women currently receive successful treatment at the lowest possible level of care. Following this explorative study, we recommend specific research into the extent of the observed gender effect. Qualitative research would be suited to improve understanding of our findings.

## Data Availability

The datasets generated and analyzed during the current study are not publicly available because they contain information that could compromise the privacy of research participants, but are available from the corresponding author upon reasonable request. The dataset that was used to provide demographic information in this study (“Bydelsinfo” from Oslo municipality) is available in a public repository that does not issue datasets with DOIs [23].

## Abbreviations

Ahus: Akershus University Hospital
CI: Confidence Interval
GP: General Practitioner
GLMM: Generalized Linear Mixed Model
ICPC: International Classification of Primary Care
Mental Health Problem (MHP); NAV: the Norwegian Labor and Welfare Administration
OR: Odds Ratio
SMHC: Specialized Mental Health Care
